# Endoscopy-assisted laparoscopic wedge-resection of gastric glomus tumor: A case report

**DOI:** 10.1016/j.ijscr.2024.110100

**Published:** 2024-08-03

**Authors:** Jozyel Castro Cláudio, Paulo Antonio Martins Filizzola, Higino Felipe Figueiredo, Daniel Lourenço Lira, Aline Pereira da Costa, Tiago Magalhães Cardoso

**Affiliations:** a Department of General Surgery of Samel Hospital, M Street, 110c, Compensa, 69030-360 Manaus, Brazil; b School of Medicine of Federal University of Amazonas, Afonso Pena Street, 1053, Centro, 69020-160 Manaus, Brazil; c Department of Endoscopy of Medinova Gastrocentro, Umberto Calderaro Avenue, 455, Room 1410, Adrianópolis, 69057-015 Manaus, Brazil

**Keywords:** Glomus tumor, Stomach neoplasm, Laparoscopy, Endoscopy, Case report

## Abstract

**Introduction:**

Glomus tumor is a pericytic mesenchymal neoplasm that most commonly occurs in the extremities. The occurrence in visceral organs is rare and is a differential diagnosis with other gastric submucosal tumors.

**Presentation of case:**

A woman with epigastric pain underwent esophagogastroduodenoscopy (EGD) which revealed a gastric submucosal tumor. Endoscopic ultrasound with fine-needle aspiration allowed preoperative diagnosis of gastric glomus tumor. Intraoperative EGD-assisted laparoscopic segmental gastrectomy was successfully performed. The patient was discharged in the second postoperative day. There was no evidence of recurrence at 8 months of follow-up.

**Discussion:**

The stomach is a rare location for the glomus tumor, a neoplasm of the glomus body, which is a perivascular structure with thermoregulatory function. Preoperative diagnosis is challenging, and endoscopic ultrasound (EUS) is useful for both assessing malignancy-associated features and biopsy guiding. The treatment is surgical resection with attention to adequate oncological margins while preserving healthy gastric wall.

**Conclusion:**

Immunohistochemical analysis of specimen obtained by EUS fine-needle allows accurate preoperative diagnosis and laparoscopic-endoscopic combined surgery allows good oncological and functional results.

## Highlights

• Gastric glomus tumor is rare submucosal tumor.
• Preoperative diagnosis is challenging, and endoscopic ultrasound may be helpful.
• Adequate preoperative assessment permits a minimally invasive approach.
• Laparoscopic-endoscopic cooperative surgery (LECS) can be oncologically safe.

## 1 Introduction

Glomus tumor (GT) is a rare benign mesenchymal neoplasm originating from the thermoregulatory structure called the glomus body and is composed of modified smooth muscle cells [1]. It is most found in the soft tissues of extremities, notably in the subungual bed, but it can occur anywhere in the body [2] and was first described in the stomach by Kay et al. in 1951 [3]. Since then, <200 cases of gastric glomus tumor (GGT) have been reported [4,5]. Here we report the case of a woman with gastric glomus tumor causing epigastric pain, who presented at a private hospital, diagnosed preoperatively, and treated with endoscopy-assisted laparoscopic resection. This work is reported in line with the SCARE 2023 criteria [6].

## 2 Presentation of case

A 56-year-old woman from the Brazilian Amazon sought medical care in a private hospital due to epigastric pain with a history of chronic venous insufficiency and no other comorbidities. She had no previous personal history of neoplasms, smoking or alcohol consumption and no relevant cancer family history. There were no findings on physical examination.

**Fig. 1:**
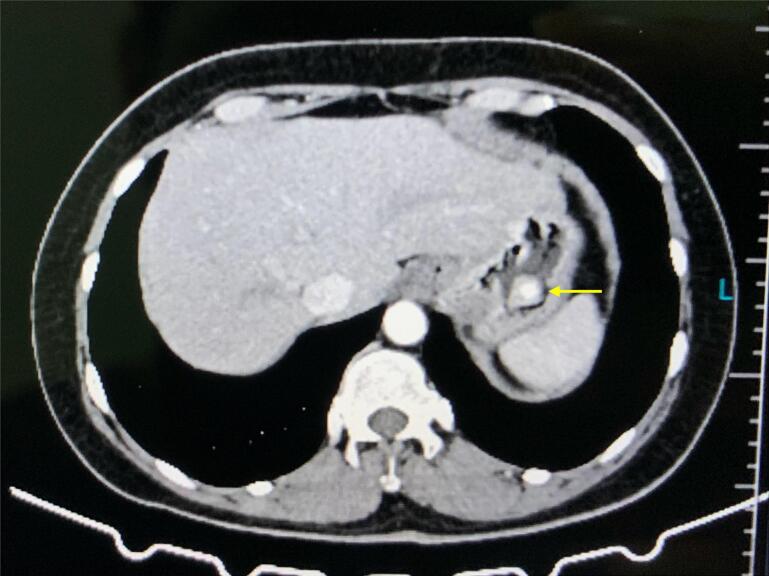
Abdominal CT with a solitary gastric lesion (arrow) and a hypervascular enhancement on arterial phase.

In the investigation of the abdominal pain, an esophagogastroduodenoscopy (EGD) was performed, which identified an elevated lesion in the gastric fundus-body transition, and a biopsy was performed. Abdominal computed tomography (CT) did not present any additional lesions (Fig. 1).

Microscopy revealed a mesenchymal neoplasm, with clusters of smooth muscle cells and blood vessels, moderate cellularity, rare mitoses and absence of atypical mitotic figures or necrosis in hematoxylin and eosin staining. Immunohistochemistry (IHC) showed positivity for smooth muscle actin, type IV collagen and Ki-67 index of 2 %, and negativity for KIT, CD34, desmin, chromogranin A, S100, synaptophysin and DOG1, favoring the diagnosis of glomus tumor.

**Fig. 2:**
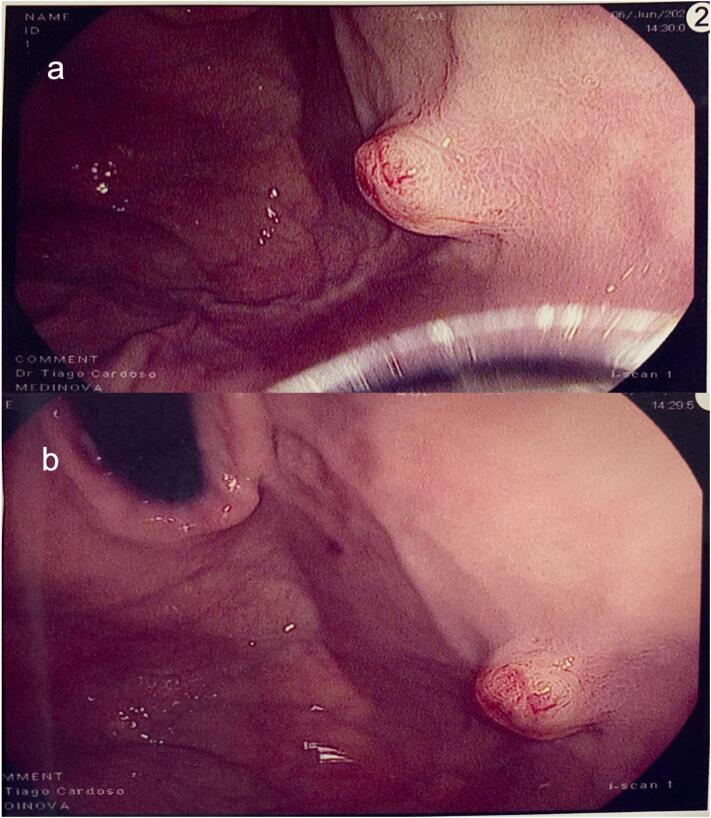
Gastric submucosal tumor at the fundus-body transition (a) and its relationship with the cardia (b) on esophagogastroduodenoscopy.

**Fig. 3:**
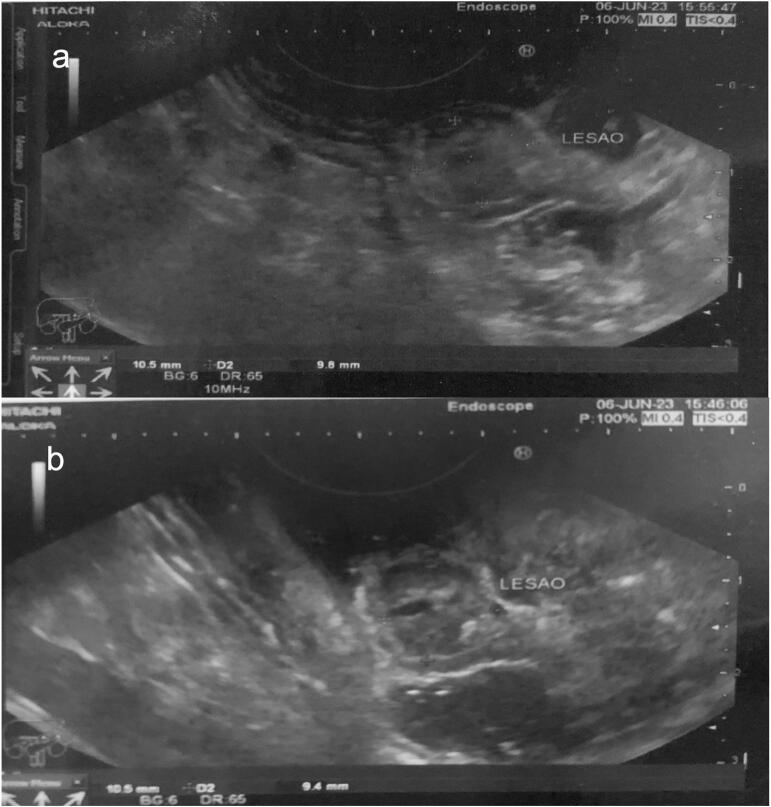
Echographic aspect of gastric submucosal tumor measuring 10.5 mm × 9.8 mm (a) and central anechoic area (b).

Endoscopic ultrasound (EUS) was performed to preoperatively mark the lesion through endoscopy for resection, and exposed an elevated, ulcerated, and irregular hypoechoic submucosal tumor (SMT), with a central anechoic area of probable origin in the fourth ultrasound layer, measuring 10.5 mm × 9.8 mm (Fig. 2, Fig. 3).

**Fig. 4:**
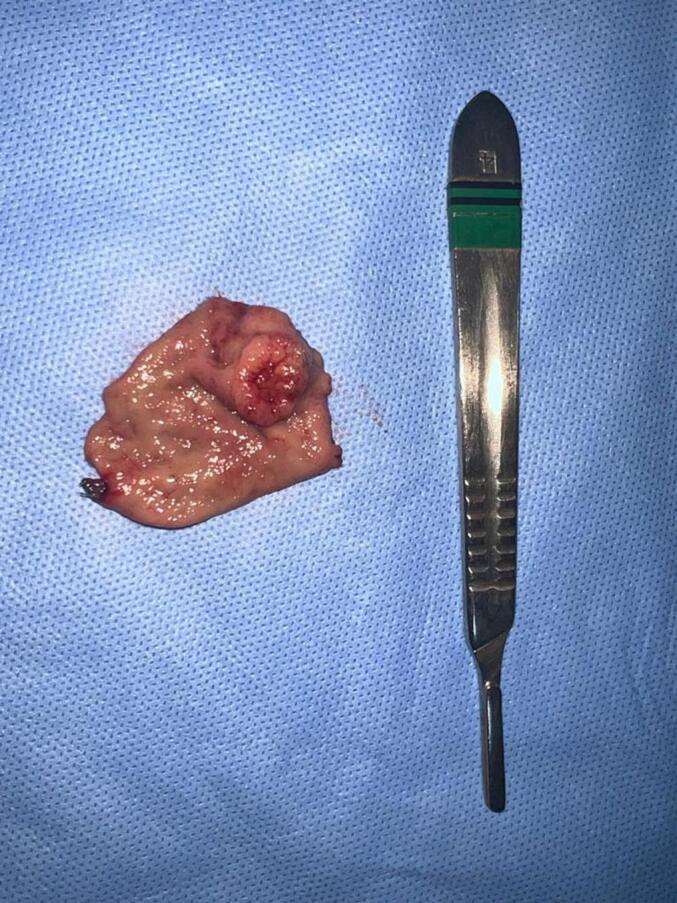
Gastric glomus tumor after wedge resection.

Due to the small size and the intraluminal component, we chose to perform laparoscopic-endoscopic cooperative surgery. Laparoscopic segmental gastrectomy was performed, assisted by a new intraoperative EGD, due to loss of previous endoscopic marking, to confirm the location of the lesion. We performed an outlining of the lesion with LigaSure followed by wedge resection using a 60 mm-endostapler with macroscopic margins of 1 cm (Fig. 4). No lymphadenectomy was performed. The operative time was 180 min, and the estimated blood loss was 20 ml. Extemporaneous pathological analysis of the surgical specimen confirmed gastric glomus tumor with free margins, with no need for additional therapy.

Postoperative course was uneventful, and the patient was discharged on the 2nd postoperative day. The patient is disease-free at 8-month follow-up with office visits every 4 months, asymptomatic and without late postoperative complications.

## 3 Discussion

The glomus body (GB) is a perivascular structure whose function is thermoregulation. It is composed of an afferent arteriole, a Sucquet-Hoyer anastomotic canal, an efferent arteriole, the intraglomerular reticulum and its capsule. A Sucquet-Hoyer canal is internally lined by endothelial cells, which are covered by smooth muscle cells, and these are interposed by glomus cuboidal cells [7]. Thus, three main structures compose glomus bodies: glomus cells, the vasculature and smooth muscle cells [7], also called pericytes, giving the nomenclature of pericytic neoplasia to GT, since the epithelioid cells, characteristic of this tumor, are morphologically equivalent to pericytic cells [3,8].

GT is usually a benign neoplasm in which there is proliferation of these components, and can be classified as solid GT, glomangioma or glomangiomyoma, depending on which component is predominantly hyperplasic [9]. Like the GB, the GT also has greater distribution in extremities [1], however, it has already been described in several extradigital sites such as the uterus, kidneys, colon, choroid, bones, muscles, trachea and mediastinum [10]. Gastric glomus tumor (GGT) preferentially affects the antrum [8] with only 5 % occurring in the fundus [4]. In the English literature, there are <200 described cases of GGT, with a predominance of women (60 %) over men and occurring on average in the 5th decade of life [4].

GGT has no specific clinical or endoscopic characteristics [11]. Epigastric pain is the most common symptom, in up to 36 % of cases, followed by upper gastrointestinal bleeding. Other clinical presentations include nonspecific abdominal pain, anemia, and diarrhea, although patients are often asymptomatic [[12], [13], [14]].

Preoperative diagnosis is challenging due to its deep location in the gastric wall, generally in the fourth ultrasound layer, corresponding to the muscularis propria [15]. However, EUS is helpful in the differential diagnosis of other SMT, identification of echographic findings associated with malignancy (heterogeneous texture, size >3 cm, irregular margins), and to perform fine-needle biopsy, allowing IHC analysis. However, there is no standardized malignant characteristics, hindering recommendations or protocols [16]. Typically, GTs present as solitary hypervascular lesions measuring up to 4 cm, with intense and persistent enhancement in the arterial and portal phases on contrast-enhanced tomography of the abdomen [8].

Microscopically, GTs present nodules of tumor cells with dilated blood vessels, separated by bundles of smooth muscle and fibrotic tissue. Some tumors have a pronounced vascular pattern, with large cavernous vessels inside. The cells have well-defined membranes and round or oval central nuclei, with eosinophilic cytoplasm. Mitoses are usually rare and sparse and nuclear atypia is generally mild [12,13]. The IHC pattern of GGTs is like soft tissue GT, expressing alpha-SMA, vimentin, collagen type IV, calponin and laminin. GGTs are negative for CD117 (KIT), CD34 (may be focally positive), desmin, cytokeratin, S-100 and DOG1 proteins [1,2,12,13].

Despite the benign course in most cases, criteria proposed by Folpe et al. defines a GT as malignant when there are atypical mitotic figures or high nuclear grade [17]. Previously considered a malignant characteristic, deep location or size >2 cm classifies GT as uncertain malignant potential, as well as an infiltrative growth pattern, high cellularity, or other atypical characteristics other than nuclear pleomorphism [9]. In our case, the microscopy with moderate cellularity, no atypical figures, rare mitoses and a small superficial tumor rule out malignancy.

The treatment of GGT is resection. Although there are reports of oncological follow-up without surgical intervention, there are still no established protocols for expectant management [16]. Gastric wedge resection is possible due to its benign nature in most cases [11], especially when the tumor is not located at the gastric inlet or outlet [4]. Laparoscopic approach is preferred and complete resection with clear margins is oncologically appropriate, with no need for lymphadenectomy. Endoscopic resection is also a possibility, although there are few reports and complications as perforation and uncontrolled bleeding may occur. In this case, surgeon's experience favored a laparoscopic approach. Chemotherapy is reserved for metastatic disease and there are no defined standardized protocols, as there is still limited data on the risk of metastasis due to shortage of reported cases of malignant GGT [4]. To date, there are no validated criteria to define resection approach based on tumor pathology.

The laparoscopic-endoscopic cooperative surgery (LECS) approach allows the identification of the margins of gastric stromal tumors and minimizes the resection of the healthy gastric wall, being originally indicated for small tumors located near the cardia and pylorus [18]. In our case, due to the small size and the intraluminal component, we chose to perform LECS. To our knowledge, this is only the sixth case described in Brazil and the first to be treated with laparoscopic wedge resection marked by endoscopy.

## 4 Conclusion

Gastric glomus tumor is a rare mesenchymal neoplasm to be considered in the investigation of gastric submucosal lesions. Preoperative immunohistochemical diagnosis of a specimen obtained with the aid of endoscopic ultrasound allows for appropriate surgical planning. The endoscopy-assisted laparoscopic approach provides adequate oncological margins through a minimally invasive technique and good functional outcome.

## Informed consent

An informed consent form was obtained from the patient.

## Ethical approval

Ethical clearance is not required for this case report, according to our institution's research ethics committee. We report the case of a single patient presenting with a rare condition, from which have obtained informed consent to report the case.

## Funding

This study has no funding sources.

## Author contribution

Jozyel Cláudio: Project administration (lead); investigation (lead); writing - reviewing and editing (equal). Paulo Filizzola: Data curation; methodology (lead); writing - original draft preparation (lead); writing - reviewing and editing (equal). Aline Costa: Visualization (lead); writing - reviewing and editing (equal). Tiago Cardoso: Investigation (supporting); writing - reviewing and editing (equal). Daniel Lira: Supervision (supporting); writing - reviewing and editing (equal). Higino Figueiredo: Supervision (lead); writing - reviewing and editing (equal).

## Guarantor

Jozyel Castro Cláudio

## Research registration number

This study is not a ‘First in Man’ trial.

## Conflict of interest statement

All authors declare having no competing interests.
